# Symptomatology and Relationship Between Symptoms and Duration Among COVID-19 Patients in a COVID-19 Care Hospital in Central India

**DOI:** 10.7759/cureus.21541

**Published:** 2022-01-24

**Authors:** Kiran Kumar Prathipati, Meena Mishra, Bharatsing Rathod, Jaya P Tripathy, Shrikrishna B H, Vijay Bidkar, Sandeep Dabhekar, Vishal Shete, Deepa G

**Affiliations:** 1 Ear, Nose, and Throat, All India Institute of Medical Sciences, Nagpur, IND; 2 Microbiology, All India Institute of Medical Sciences, Nagpur, IND; 3 General Internal Medicine, All India Institute of Medical Sciences, Nagpur, IND; 4 Preventive Medicine, All India Institute of Medical Sciences, Nagpur, IND; 5 Otolaryngology – Head and Neck Surgery, All India Institute of Medical Sciences, Nagpur, IND; 6 Anatomy, Navodaya Medical College, Raichur, IND

**Keywords:** pandemic, duration, symptoms, coronavirus, covid-19

## Abstract

The COVID-19 pandemic has affected the world population across the globe. India has the second^ ^largest number of cases and the third^ ^largest number of deaths due to COVID-19 in the world. There have been close to 4.4 lakh deaths due to COVID-19 in India alone. The second wave in India has led to devastating consequences, particularly among the young population. The initial clinical symptoms of COVID-19 are similar to all types of viral pneumonia, with varying degrees of severity. The cases’ clinical manifestations include fever, nonproductive cough, dyspnea, myalgia, and fatigue. This study was undertaken with the purpose of identifying the relationship between the symptoms and duration in COVID-19-affected patients. The common presenting symptoms were fever (44.5%), sore throat (38.7%), and cough (36.12%). Most of the cases presented with a combination of fever with cough (35%) and fever with sore throat (33%). The duration of symptoms varied from one to 17 days with a mean of 5.75 days. Despite vaccination being started, the risk of the imminent third wave in the country is existential. Mutations in the coronavirus pose a threat to the vulnerable population. It is important to identify the combination of symptoms most predictive of COVID-19 to help guide recommendations for self-isolation, testing, and preventing further spread of the disease. Further studies using these models can yield better results in surveillance and containing this infectious disease.

## Introduction

The COVID-19 pandemic has affected the world population across the globe. Globally, as of September 3, 2021, there have been 218,946,836 confirmed cases of COVID-19, including 4,539,723 deaths [1]. The first coronavirus case was reported in India on January 30, 2020, in the state of Kerala. The second wave in India has led to devastating consequences, particularly among the young population. Studies have identified various circulating double mutant and triple mutant strains of SARS-CoV-2 across different regions of India, which are more pathogenic than the initial strains. Such altered transmissibility and pathogenicity indicate the evolution of the virus [2]. Many variants have been identified in South Africa (known as 20H/501Y or B.1.351), Brazil (P.1), and the UK (B.1.1.7), alongside a distinct Indian variant (B.1.617). The B.1.617 variant has become the dominant strain across India and has spread to about 40 nations [3]. India launched its vaccine drive against COVID-19 on January 16, 2021, mostly relying on Covishield, a version of the Oxford-AstraZeneca vaccine produced by the Serum Institute of India [4].

The initial clinical symptoms of COVID-19 are similar to all types of viral pneumonia, with varying degrees of severity. The cases’ clinical manifestations include fever, nonproductive cough, dyspnea, myalgia, and fatigue. Organ dysfunction (e.g., shock, acute respiratory distress syndrome (ARDS) [5], acute cardiac injury, and acute kidney injury) and death can occur in severe cases. Age and the presence of comorbid illnesses increase the risk of death among persons with COVID-19. Data from published epidemiology and virologic studies provide evidence that COVID-19 is primarily transmitted from symptomatic people to others who are in close contact through respiratory droplets, by direct contact with infected persons, or by contact with contaminated objects and surfaces [6].

The gold standard for the etiological diagnosis of SARS-CoV-2 infection is (real-time) reverse transcription-polymerase chain reaction (RT-PCR) on respiratory tract specimens. Nasopharyngeal swab and oropharyngeal swab are used to identify persons infected with SARS-CoV-2 infection [7]. In general, the highest viral loads are observed at the time of symptom onset and for a few days after [8], with levels slowly decreasing over the next one to three weeks. There is no consensus as to the ideal time since the onset of symptoms to take the sample for testing for COVID-19. Failure to amplify can be interpreted as a negative result [9] but could also be attributable to poor quality of the clinical sample or to early disease status or severely infected cases.

Various studies state the incubation period to be between eight and 13 days [10]. The correct estimate of incubation is sometimes difficult as some cases are asymptomatic in the initial period after infection. The correct time of collection of samples is based on the incubation period. A better understanding of COVID-19-related symptoms will help optimize diagnostic strategies, especially with emerging strains. This study was undertaken with the purpose of identifying the relationship between the symptoms and duration in COVID-19-affected patients.

## Materials and methods

Study design and study population

A cross-sectional study was conducted in a tertiary care hospital among suspected patients of COVID-19. The study was approved by the institutional ethical committee. Written informed consent was undertaken from all patients. All those who presented to the screening outpatient department (OPD) from May 2020 were assessed according to the Indian Council of Medical Research (ICMR) guidelines for testing for COVID-19 [11,12].

Details of epidemiological significance (age, sex, and history of international travel), dates of exposures, and symptom onset were recorded. The participants’ swabs were taken from the nasopharynx and oropharynx and sent to the laboratory for COVID-19 testing with RT-PCR. Data were extracted from the specimen referral form taken from the COVID-19 laboratory [13].

The inclusion criteria of the study were in accordance with the ICMR guidelines dated April 9, 2020, which were updated on May 18, 2020 [11,12], and include the following: all symptomatic individuals who have undertaken international travel in the last 14 days (category 1), all symptomatic contacts of laboratory-confirmed cases (category 2), all symptomatic healthcare workers (category 3), all cases with severe acute respiratory illness (SARI) (category 4), asymptomatic direct and high-risk contacts of a confirmed case in hotspots/clusters (as per the Ministry of Health and Family Welfare (MoHFW)) and in large migration gatherings/evacuee centers (category 5), all symptomatic influenza-like illness (ILI) (including fever, cough, sore throat, and runny nose) (category 6), all hospitalized patients who develop ILI symptoms (category 7), and all symptomatic ILI among returnees and migrants within seven days of illness (category 8).

ILI case is defined as one with acute respiratory infection with fever ≥ 38°C and cough. SARI case is defined as one with acute respiratory infection with fever ≥ 38°C and cough and requiring hospitalization.

The participants whose oropharyngeal and nasopharyngeal swab report was inconclusive were excluded from the study.

**Figure 1:**
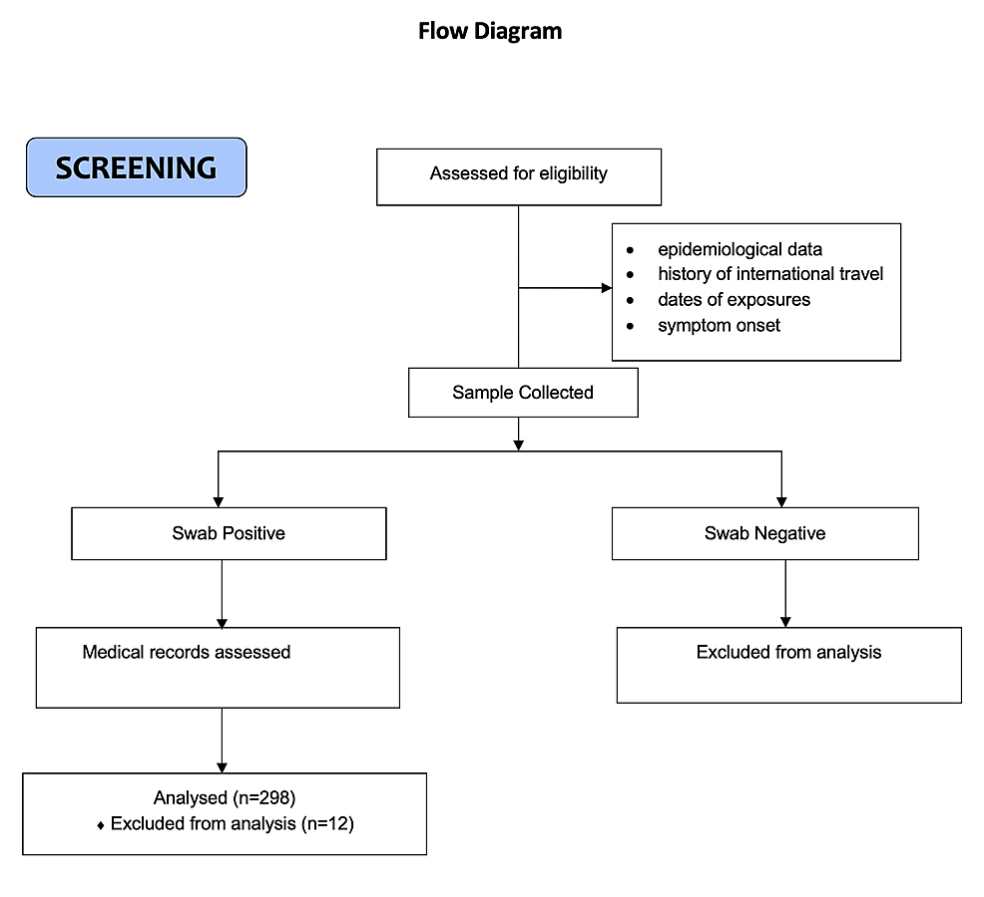
Flow diagram for the conduct of the study

Overall, 310 patients were screened for the study, and a total of 298 participants were included in the study. Twelve cases were excluded due to incomplete data. The flow diagram for the study is given in Figure 1.

Statistical analysis

All statistical analyses were conducted using SPSS version 20.0 (IBM Corp., Armonk, NY, USA). Continuous variables were expressed as means and standard deviation (SD) when normality criteria were met, otherwise as median and ranges. Categorical variables were reported as numbers and percentages. The Chi-square test was used to compare categorical variables with Fisher’s exact test, as needed. Pearson’s r correlation was used to correlate continuous variables. Statistical significance level was taken as p < 0.05 (two-sided).

## Results

**Figure 2:**
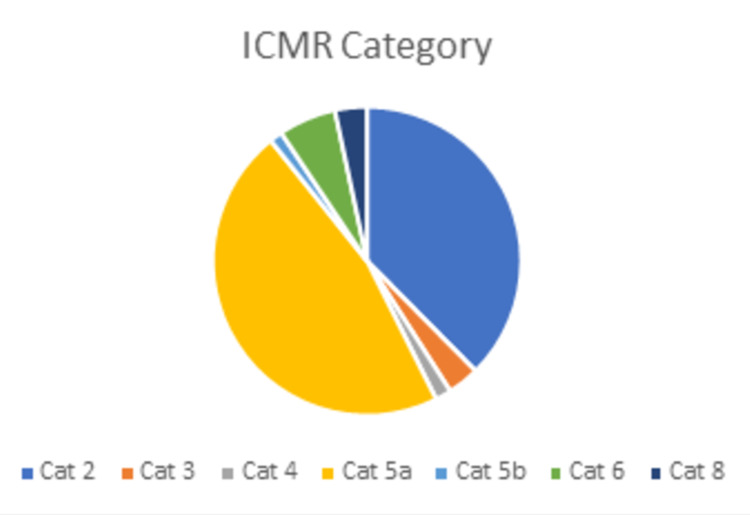
Distribution of cases according to the ICMR categories Cat 1: Symptomatic international traveler in last 14 days Cat 2: Symptomatic contact of a laboratory-confirmed case Cat 3: Symptomatic healthcare worker Cat 4: Severe acute respiratory illness (SARI) patient Cat 5a: Asymptomatic direct and high-risk contact of a laboratory-confirmed case Cat 5b: Asymptomatic healthcare worker in contact with a confirmed case without adequate protection Cat 6: Symptomatic influenza-like illness (ILI) patient in MoHFW-identified clusters Cat 8: Symptomatic influenza-like illness among returnees and migrants within seven days of illness Cat: category

Between May 25 and July 31, 2020, 298 adult patients were tested with suspicion of COVID-19 in our institution. A total of 298 patients who had positive tests for SARS-CoV-2 RNA who met the inclusion criteria were enrolled in the study. We applied the Strengthening the Reporting of Observational Studies in Epidemiology (STROBE) guidelines for reporting. The patients’ mean age was 32 years, with most cases being adults. Of them, the majority were males (182, 61.14%). Of the total, 143 (47.97%) cases were asymptomatic (95% CI = 42.2-53.8). Asymptomatic direct and high-risk contact of the laboratory-confirmed cases (category 5a according to the ICMR guidelines) formed the most common group, with 139 (46.64%) patients. Nineteen (6.41%) patients had a prior history of travel. Ten (3.4%) healthcare workers were found to be positive. The age and demographic profile of the COVID-19 patients are mentioned in Table 1. The distribution of cases according to the ICMR categories is displayed in Figure 2.

**Table 1:** Demographic characteristics of the COVID-19 patients

Total (N = 298)	Number (%)
Gender	
Male	116 (39)
Female	182 (61)
Age	
0–14 years	43 (14.4)
15–44 years	185 (62)
45–59 years	55 (18.5)
60 and above	14 (4.7)

**Figure 3:**
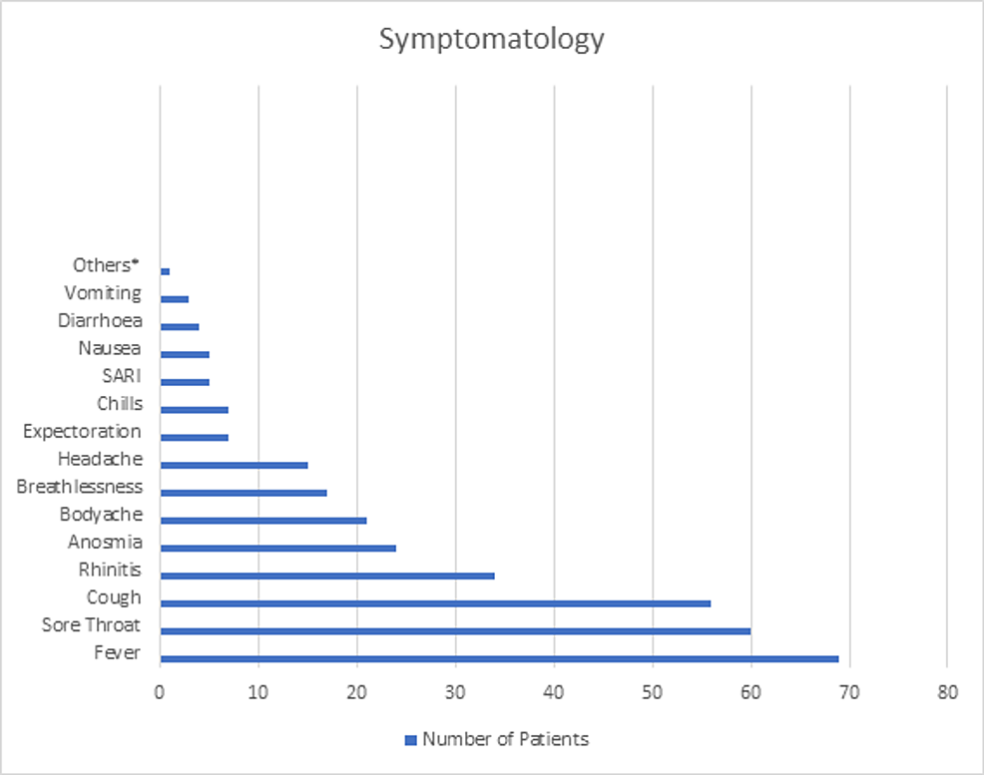
Distribution of symptoms of the COVID-19 patients *Others: ear discharge, generalized weakness, fatigue, rash, abdominal pain

The majority of the cases were tested within one week of symptom onset. The third day has the highest rate (73.6%) of the time point when participants tested positive. Most cases (73.6%) were found to be positive within three days of symptom onset. The common presenting symptoms were fever (44.5%), sore throat (38.7%), and cough (36.12%). Most of the cases presented with a combination of fever with cough (35%) and fever with sore throat (33%). Five (3.2%) cases presented with severe acute respiratory illness (SARI). The duration of symptoms varied from one to 17 days with a mean of 5.75 days (SD = 5.95). The distribution of various symptoms is shown in Figure 3.

**Figure 4:**
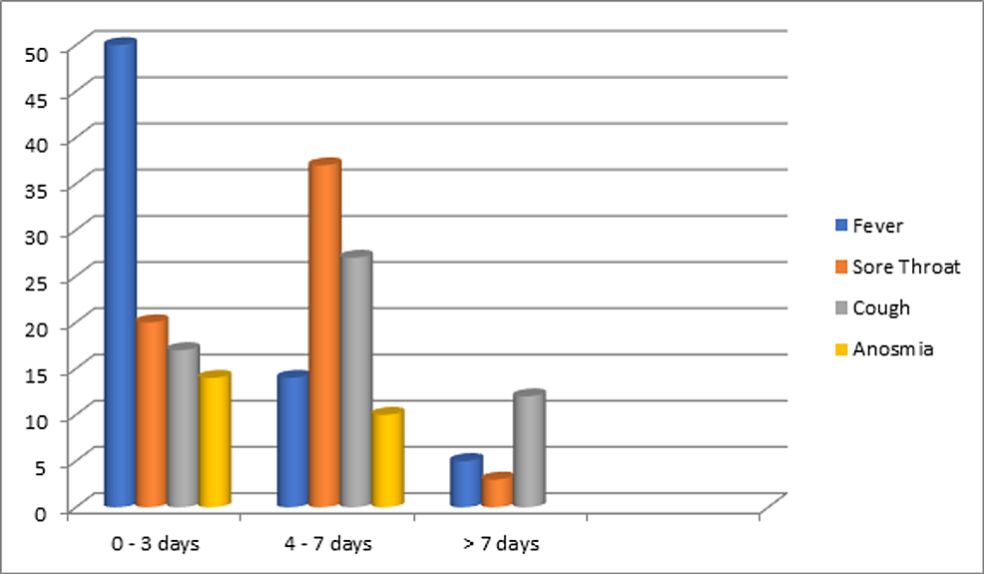
Duration of symptoms of the COVID-19 patients

The duration of symptoms is depicted in Figure 4. 

Among those patients suffering from fever, 10% of the patients had chills, 35% of the patients had a cough, and 33% of the patients had a sore throat, and this association was statistically significant (p < 0.05). Among those patients suffering from cough, 14% of the patients had breathlessness and 10% of the patients had expectoration, and this association was statistically significant (p < 0.05). Among those patients suffering from sore throat, 25% of the patients had rhinitis and 15% of the patients had anosmia, and this association was statistically significant (p < 0.05). Seven patients had rhinitis with anosmia, and this association was statistically significant (p < 0.05). The association of fever with other symptoms is shown in Table 2.

**Table 2:** Common symptom groups associated with fever (N = 69)

	n	%	p-value
Fever with chills	7	10%	0.000
Fever with cough	24	35%	0.000
Fever with sore throat	23	33%	0.003
Fever with breathlessness	9	13%	0.006
Fever with expectoration	2	2.9%	0.66
Fever with diarrhea	3	4.3%	0.04
Fever with nausea	1	1.4%	1.000
Fever with vomiting	1	1.4%	0.54
Fever with rhinitis	10	14.5%	0.389
Fever with body ache	8	11.8%	1.10
Fever with headache	3	4.4%	1.000
Fever with anosmia	5	7.2%	1.000
Fever with SARI	3	4.3%	0.08

The association of cough with other symptoms is shown in Table 3.

**Table 3:** Common symptom groups associated with cough (N = 56)

	n	%	p-value
Cough with chills	3	5.4%	0.12
Cough with sore throat	16	28.6%	0.09
Cough with breathlessness	8	14.3%	0.006
Cough with expectoration	6	10.7%	0.000
Cough with diarrhea	3	5.4%	0.02
Cough with nausea	1	1.8%	1.000
Cough with vomiting	1	1.8%	0.466
Cough with rhinitis	11	19.6%	0.059
Cough with body ache	9	16.4%	0.007
Cough with headache	2	3.6%	1.00
Cough with anosmia	4	7.1%	1.00
Cough with SARI	3	5.4%	0.04
Cough with ear discharge	1	1.8%	0.18

The association of sore throat with other symptoms is shown in Table 4. 

**Table 4:** Common symptom groups associated with sore throat (N = 60)

	n	%	p-value
Sore throat with chills	2	3.3%	0.63
Sore throat with breathlessness	3	5%	1.00
Sore throat with expectoration	4	6.7%	0.03
Sore throat with diarrhea	2	3.3%	1.18
Sore throat with nausea	3	5%	0.057
Sore throat with vomiting	2	3.3%	0.10
Sore throat with rhinitis	15	25%	0.001
Sore throat with body ache	7	11.9%	0.15
Sore throat with headache	4	6.7%	0.51
Sore throat with anosmia	9	15%	0.03
Sore throat with SARI	1	1.7%	1.00

The association of rhinitis with other symptoms is shown in Table 5. 

**Table 5:** Common symptom groups associated with rhinitis (N = 34)

	n	%	p-value
Rhinitis with chills	1	2.9%	0.56
Rhinitis with breathlessness	0	0%	0.23
Rhinitis with expectoration	1	2.9%	0.57
Rhinitis with diarrhea	1	2.9%	0.38
Rhinitis with nausea	2	5.9%	0.10
Rhinitis with vomiting	1	2.9%	0.30
Rhinitis with body ache	3	8.8%	0.71
Rhinitis with headache	2	5.9%	0.68
Rhinitis with anosmia	7	20.6%	0.01

## Discussion

India has the second largest number of cases and the third largest number of deaths due to COVID-19. There have been close to 4.4 lakh deaths due to COVID-19 in India alone [1]. Despite vaccination being started, the risk of the imminent third wave in the country is existential. Mutations in the coronavirus pose a threat to the vulnerable population. India has a large and diversified population, and fighting this pandemic is difficult because of the overburdened health sector and financial constraints. It is imperative to know the common symptoms of presentation of this infectious condition. To contain the spread of this virus, it is essential to recognize the symptoms early and get the cases tested for COVID-19.

In India, the ICMR issued guidelines for testing symptomatic international travelers in March 2020 [14], which was subsequently expanded to include both symptomatic individuals and high-risk contacts of confirmed cases. The B.1.617.2, a variant of COVID-19 known as the Delta variant, was first identified in October 2020 in India [15] and was primarily responsible for the second wave in the country. There is an increased risk of virus transmission because of relaxed norms of isolation and quarantine. There is an increased need to analyze and stratify cases according to the symptoms so as to pick up these infections early.

The common symptoms of COVID-19 found in our study are fever (44.5%), cough (38.7%), and sore throat (36.12%). The prevalence of fever was 78% and cough (57%) in the study done by Grant et al. [16]. The frequency of symptoms in our study is less compared to studies done by Grant et al., Larsen et al., Menni et al., and Perlman et al. [16-20]. In our study, the duration of symptoms varied from one to 17 days, with a mean of 5.75 days (SD = 5.95). With respect to the correlation of the duration of symptoms with RT-PCR positivity, there is no statistically significant correlation between the duration of symptoms and RT-PCR positivity (p = 0.114), although there is a clinical correlation between the two variables. Studies assessing the duration of symptoms show a mean duration of eight days (6.25-11.5) [21,22]. The participants reported a median of three days (IQR: 2-4) of symptoms before SARS-CoV-2 test in the study done by Fisher et al., which is similar to our study [21]. Frequently, the cases present with a combination of fever, cough, and sore throat. This is similar to the results observed in the meta-analysis done by Grant et al. [16]. Limited studies have taken into consideration these combinations of symptoms. The common combination of symptoms seen in our study was fever with sore throat (35%), fever with cough (33%), and sore throat with cough (28.6%). Fever and body aches, weakness, or fatigue were also more likely to be reported among cases than controls in the study done by Fisher et al. [21].

A combination of loss of smell and taste, fatigue, persistent cough, and loss of appetite resulted in the best model in the study by Larsen et al. [17]. Further evaluation of these combinations of symptoms can yield better predictive results. Loss of smell was present in 24 (8.1%) cases. The prevalence of loss of smell and taste was threefold higher in individuals who tested positive (65.03%) than in those who tested negative (21.71%) in the study by Larsen et al. [17]. Recent onset loss of smell and taste is specific to COVID-19 [17], even in the absence of fever and cough. Very few cases had gastrointestinal symptoms such as nausea, vomiting, and abdominal pain (4.3%). This is similar to the meta-analysis done by Mao et al. [23]. The newer variants of the virus have caused more serious symptoms, and more extrapulmonary symptoms have been reported in the second wave of the pandemic [15].

## Conclusions

It is important to identify the combination of symptoms most predictive of COVID-19, to help guide recommendations for self-isolation, testing, and preventing further spread of the disease. Further studies may be required to see if the symptoms vary according to various strains of the virus and also if there is any variation in relation to risk factors such as obesity, previous heart disease, and COPD. Future studies using these models can yield better results in surveillance and containing this infectious disease. The newer variant of coronavirus, Omicron, which originated from South Africa, has reached many counties with the possibility of a newer wave of the pandemic. Hence, every effort should be made for a structured reporting of symptoms and surveillance of this variant.

## Appendices

The symptoms of the patients with COVID-19 are depicted in Table 6.

**Table 6:** Symptoms of the COVID-19 patients

Symptoms	n	%	95% confidence interval
Fever	69	23.2	18.5–28.4
Chills	7	2.3	1.0–4.8
Cough	56	18.8	14.5–23.7
Sore throat	60	20.1	15.7–25.1
Breathlessness	17	5.7	3.4–9.0
Expectoration	7	2.3	1.0–4.8
Diarrhea	4	1.3	0.4–3.4
Nausea	5	1.7	0.6–3.9
Vomiting	3	1	0.2–2.9
Abdominal pain	1	0.3	0.01–1.86
Rhinitis	34	11.4	8.0–15.6
Bodyache	21	7	4.4–10.6
Headache	15	5	2.8–8.2
Anosmia	24	8.1	5.2–11.8
SARI	5	1.7	0.6–3.9
Ear discharge	1	0.3	0.01–1.86
Generalized weakness	1	0.3	0.01–1.86
Fatigue	1	0.3	0.01–1.86
Rash	1	0.3	0.01–1.86
